# Occurrence, Risk Factors, and Outcomes of Pulmonary Barotrauma in Critically Ill COVID-19 Patients: A Retrospective Cohort Study

**DOI:** 10.1155/2023/4675910

**Published:** 2023-02-22

**Authors:** Hasan M. Al-Dorzi, Haifa Almujaydia, Reema Nazer, Yara Alhusaini, Aminah Alhamdan, Ajyad Al Jawad

**Affiliations:** ^1^College of Medicine, King Saud Bin Abdulaziz University for Health Sciences, King Abdullah International Medical Research Center and Intensive Care Department, King Abdulaziz Medical City, Ministry of National Guard Health Affairs, Riyadh, Saudi Arabia; ^2^College of Medicine, King Saud Bin Abdulaziz University for Health Sciences, Riyadh, Saudi Arabia

## Abstract

**Objective:**

Pulmonary barotrauma has been frequently observed in patients with COVID-19 who present with acute hypoxemic respiratory failure. This study evaluated the prevalence, risk factors, and outcomes of barotrauma in patients with COVID-19 requiring ICU admission.

**Methods:**

This retrospective cohort study included patients with confirmed COVID-19 who were admitted to an adult ICU between March and December 2020. We compared patients who had barotrauma with those who did not. A multivariable logistic regression analysis was performed to determine the predictors of barotrauma and hospital mortality.

**Results:**

Of 481 patients in the study cohort, 49 (10.2%, 95% confidence interval: 7.6–13.2%) developed barotrauma on a median of 4 days after ICU admission. Barotrauma manifested as pneumothorax (*N* = 21), pneumomediastinum (*N* = 25), and subcutaneous emphysema (*N* = 25) with frequent overlap. Chronic comorbidities and inflammatory markers were similar in both patient groups. Barotrauma occurred in 4/132 patients (3.0%) who received noninvasive ventilation without intubation, and in 43/280 patients (15.4%) who received invasive mechanical ventilation. Invasive mechanical ventilation was the only risk factor for barotrauma (odds ratio: 14.558, 95% confidence interval: 1.833–115.601). Patients with barotrauma had higher hospital mortality (69.4% versus 37.0%; *p* < 0.0001) and longer duration of mechanical ventilation and ICU stay. Barotrauma was an independent predictor of hospital mortality (odds ratio: 2.784, 95% confidence interval: 1.310–5.918).

**Conclusion:**

s. Barotrauma was common in critical COVID-19, with invasive mechanical ventilation being the most prominent risk factor. Barotrauma was associated with poorer clinical outcomes and was an independent predictor of hospital mortality.

## 1. Introduction

Critical illness may occur in patients with Coronavirus Disease 2019 (COVID-19), mainly in the form of severe pneumonia and acute hypoxemic respiratory failure, which may occur in 17–29% of hospitalized patients [1–4]. Pulmonary barotrauma, defined as the aberrant presence of gas in extraalveolar locations, thus causing pneumothorax, pneumomediastinum, and/or subcutaneous emphysema, was commonly encountered in clinical practice and then described in observational studies [5]. A systematic review of 15 observational studies of COVID-19 found barotrauma in 4.2% (95% confidence interval [CI]: 2.4–7.3%) of hospitalized patients, 15.6% (95% CI: 11–21.8%) of critically ill patients, and 18.4% (95% CI: 13–25.3%) of patients receiving invasive mechanical ventilation (IMV) [5].

COVID-19 itself may increase the risk of pulmonary barotrauma. One study observed that 89/601 patients (14.8%) who had COVID-19 and received IMV suffered from barotrauma, compared with only 1/196 patients (0.5%) who were admitted during the same period and tested negative for COVID and 31/285 patients (10.9%) with acute respiratory distress syndrome [6]. A systematic review of 13 observational studies of patients with COVID-19 receiving IMV found that 266/1814 patients (14.7%) had at least one barotrauma event compared with 31/493 patients (6.3%) with non-COVID acute respiratory distress syndrome (based on data from 3 studies) [7]. A subsequent large retrospective study found that pneumothorax/pneumomediastinum risk was significantly higher in COVID-19 (2211 patients) versus prepandemic acute respiratory distress syndrome (5522 patients) (adjusted odds ratio [OR]: 1.31, 95% CI: 1.13–1.52) [8]. Barotrauma may be related to the severity of COVID-19 and IMV [5, 6]. A retrospective study of patients with COVID-19 and acute hypoxemic respiratory failure found that barotrauma occurred in 11/232 patients (4.7%) who received noninvasive ventilation (NIV) and in 21/121 patients (17.4%) who received IMV [9]. Pulmonary barotrauma in patients with COVID-19 has been associated with increased morbidity and in-hospital mortality [5, 8].

Studies on the epidemiology of barotrauma in critical COVID-19 patients remain uncommon. Its risk factors are not well characterized, especially since many of the published studies were performed in patients receiving IMV, whereas barotrauma has been observed in other patients with COVID-19 [5]. This study evaluated the prevalence of barotrauma, risk factors, and outcomes of pulmonary barotrauma in COVID-19 patients admitted to the ICU because of acute hypoxemic respiratory failure.

## 2. Methods and Materials

### 2.1. Setting and Patients

This retrospective cohort study was conducted in King Abdulaziz Medical City in Riyadh, Saudi Arabia, a tertiary-care center with more than 1000-bed capacity. Its Intensive Care Department had seven different ICUs, four of which were designated as COVID-19 units during the pandemic [10]. The ICUs operated as closed units with 24-hour per day, 7-days per week on-site coverage by board-certified intensivists [10]. In this study, we included patients who were older than 14 years (the cutoff age for admission to an adult ward/ICU in Saudi Arabia), confirmed to have COVID-19, and admitted to the adult ICU between March 1 and December 31, 2020, because of acute hypoxemic respiratory failure that was treated by any form of oxygen therapy. A confirmed COVID-19 case was defined as one with a clinical presentation consistent with COVID-19 and detection of severe acute respiratory syndrome coronavirus 2 (SARS-CoV-2) RNA in a respiratory specimen by a real-time reverse transcription polymerase chain reaction. Critical COVID-19 was defined as having acute respiratory failure, septic shock and/or multiple organ dysfunction [11]. Patients who were transferred from other institutions with a known history of pulmonary barotrauma and those who did not undergo a chest X-ray or computed tomography were excluded. The study was approved by the Institutional Review Board of the Ministry of National Guard Health Affairs, Riyadh, Saudi Arabia.

### 2.2. Data Collection

All patients' clinical data and information were retrieved from the electronic health record system of the hospital (BESTCare). Data included socio-demographic factors such as age, gender, body mass index (BMI), chronic comorbidities (diabetes, hypertension, chronic obstructive pulmonary disease, asthma, previous pneumothorax, interstitial lung diseases), and smoking. Other collected data included chest X-ray and computed tomography findings (location of infiltrates, presence of subcutaneous emphysema and location, presence of pneumomediastinum, presence of pneumothorax and location) for the first 10 days in ICU, laboratory results (including white blood cell counts, neutrophil and lymphocyte counts, inflammatory markers, ferritin levels, and D-dimer), treatments such as vasopressors, central venous catheters, renal replacement therapy, corticosteroids and dose, use of different oxygen therapies (conventional oxygen therapy low-flow oxygen devices such as nasal prongs, mask with or without oxygen reservoir, Venturi mask systems), high-flow nasal cannula, NIV and IMV, timing of barotrauma in relationship to IMV, ventilator settings in the first 24 hours (max tidal volume, max positive end expiratory pressure (PEEP), peak pressure, and plateau pressure) for patients who received IMV, and insertion of chest tube (number and location).

The primary outcome of the study was hospital mortality. The secondary outcomes were ICU mortality, duration of mechanical ventilation, need for a tracheostomy, and length of stay in the ICU and hospital.

### 2.3. Statistical Analysis

The prevalence of pulmonary barotrauma (with 95% CI) was calculated in the study cohorts as well as in subgroups who received different forms of oxygen therapy and mechanical ventilation. The study patients were categorized into two groups: patients who developed pulmonary barotrauma and those who did not. The descriptive statistics were presented as frequency and percentage for categorical variables and as mean with standard deviation or median with the first and third quartiles (*Q*1, *Q*3) for continuous variables, depending on the normality of their distribution. Multivariable logistic regression analysis was performed to evaluate the risk factors for pulmonary barotrauma and for hospital mortality. In the models, the independent variables were those with *p* value <0.25 between groups [12]. To evaluate the risk factors for mortality associated with barotrauma, we compared the characteristics and management of patients with barotrauma who survived and did not survive. The results of the regression analyses were presented as an OR with a 95% CI. We used Statistical Package for the Social Sciences (version 21) software for statistical analysis. A test was considered significant if the *p* value was <0.05.

## 3. Results

### 3.1. Patient Characteristics

Between the 1^st^ of March of 2020 and the 31^st^ of December of 2020, a total of 481 patients with critical COVID-19 were admitted to the different ICUS. The mean age was 61.3 ± 14.8 years, and the majority (71.9%) of patients were males. The median BMI was 30.1 kg/m^2^ (interquartile range: 25.8, 34.6 kg/m^2^). Smoking history was unavailable for the vast majority of patients. The common comorbidities were hypertension (67.4%) and diabetes (82.7%). During the 10-day evaluation period, all patients had at least one chest X-ray, and 79 had chest computed tomography. Most (82.1%) patients had bilateral lung infiltrates on chest X-ray performed on the first day. Most patients received NIV (302/481, 62.8%), mostly alternating with high-flow nasal oxygen (294/481, 61.1%), and 280 (58.2%) patients required intubation and IMV, mostly after a trial of NIV.

### 3.2. Prevalence and Predictors of Pulmonary Barotrauma

Out of 481 patients, 49 (10.2%, 95% CI: 7.6–13.2%) developed barotrauma on a median of 4 days after ICU admission (interquartile range: 2, 7 days). In 5 patients, pulmonary barotrauma was diagnosed by chest computed tomography as it was not seen on a chest X-ray. The barotrauma was of different types with frequent overlap: 21 patients (42.9%) presented with pneumothorax, 25 (51%) with pneumomediastinum, and 25 (51%) with subcutaneous emphysema. Pneumomediastinum and subcutaneous emphysema without pneumothorax occurred in 12 and 14 patients, respectively.

**Figure 1:**
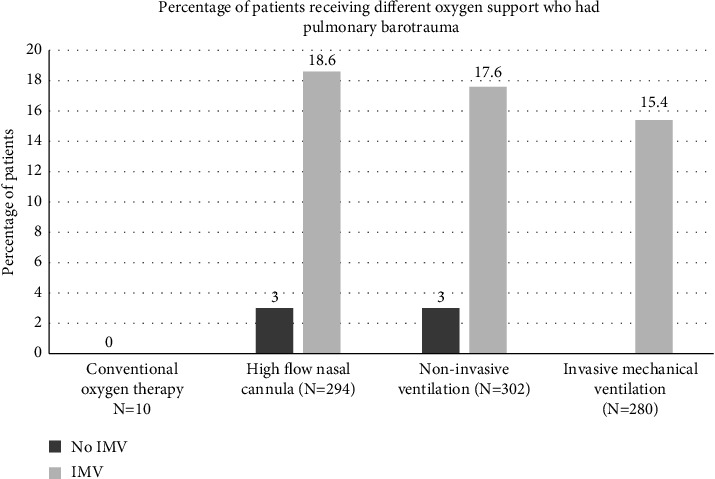
Rate of barotrauma according to the oxygen support modality. Conventional oxygen therapy alone was provided to 10 patients. High-flow nasal oxygen was provided to 294 patients (alternating with non-invasive ventilation in 208 patients); barotrauma occurred in 29 patients (5/165 who did not receive invasive mechanical ventilation (IMV) and 24/129 who received IMV). Noninvasive ventilation was provided to 302 patients; barotrauma occurred in 30 patients (4/132 who did not receive IMV and 30/170 who received IMV). IMV was provided to 280 patients; barotrauma occurred in 43 patients (8/83 patients who received solely IMV without noninvasive ventilatory support and 35/197 patients who had IMV after noninvasive ventilatory support).

The rates of barotrauma according to the different oxygen support modalities are shown in Figure 1. None of the patients who received only conventional oxygen therapy (oxygen by nasal cannula or face mask) had barotrauma. For patients who were treated with high flow nasal oxygen, barotrauma occurred in 29/294 (9.9%, 95% CI: 6.7–13.9%); in 5/165 (3.0%, 95% CI: 1.0–6.9%) who did not receive IMV; and in 24/129 (18.6%, 95% CI: 12.3–26.4%) who subsequently received IMV (*p* < 0.0001). For patients who were treated with NIV, barotrauma occurred in 34/302 (11.3%, 95% CI: 7.9–15.4%); in 4/132 (3.0%, 95% CI: 0.8–7.6%) who did not require subsequent IMV; and in 30/170 (17.6%, 95% CI: 12.2–24.2%) who required subsequent IMV (*p* < 0.0001). For patients who received IMV, barotrauma occurred in 43/280 (15.4%, 95% CI: 11.3–20.1%); 8/83 (9.6%) patients who received solely IMV without noninvasive ventilatory support; and 35/197 (17.8%) patients who had IMV after noninvasive ventilatory support (*p*=0.09). Among patients receiving IMV, most (78.4%) barotrauma events occurred after intubation, and two events were probably related to the insertion of central venous catheters.

There were no significant differences in most baseline characteristics, including presence of comorbidities, BMI, and laboratory findings, between patients with or without barotrauma (Table 1). There were no significant differences in the patterns of infiltration on CXR between the two groups. IMV was more common in patients who developed barotrauma compared to those who did not; 87.8% of patients with barotrauma were intubated compared with 54.9% of those without barotrauma (*p* < 0.0001). The mechanical ventilator settings on the first day of intubation are described in Table 1. These settings of the mechanical ventilator were not different between the two groups.

Corticosteroids, in the form of dexamethasone, hydrocortisone, and methylprednisolone, were given to 43/49 patients (87.8%) with barotrauma and 345/452 patients (79.9%) without barotrauma (*p*=0.185). For patients who received dexamethasone, the dose was significantly higher in patients with barotrauma (median of 6 mg [interquartile range: 6–12 mg] versus 6 mg [interquartile range: 6–8 mg] for patients without barotrauma; *p*=0.009).

**Figure 2:**
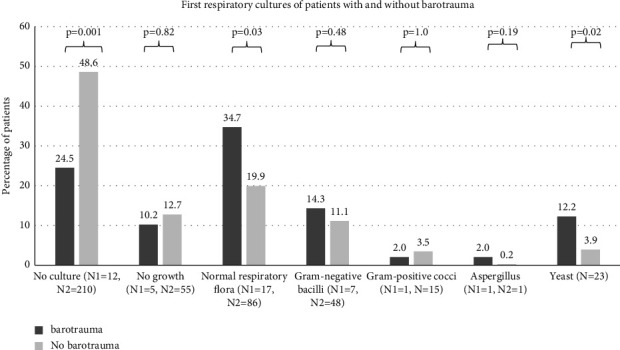
Results of respiratory bacterial cultures from patients with and without pulmonary barotrauma. *N*1 refers to the number of patients with barotrauma who had the corresponding culture. *N*2 refers to the number of patients without barotrauma who had the corresponding culture. The percentage of patients with positive cultures is shown on the *Y*-axis.

Respiratory bacterial cultures were taken in 222 patients. Their results are shown in Figure 2. More patients with barotrauma had respiratory cultures than those without barotrauma. Normal respiratory flora and yeast were isolated more often in patients with barotrauma.

Males and females had similar rates of barotrauma (37/346 (10.7%) patients and 12/135 (8.9%) patients, respectively; *p*=0.56). On multivariable logistic regression analysis (Table 2), in which obesity, diabetes, asthma, hyperlipidemia, cancer, lymphocyte count, lactate level, D-dimer, IMV, and systemic steroids were entered in the model as independent variables, only IMV was significantly associated with barotrauma (OR: 14.558, 95% CI: 1.833–115.601).

### 3.3. Management of Barotrauma

Most patients with barotrauma were treated conservatively, as chest tube insertion was performed in 21/49 patients (42.9%); bilaterally in 9 patients; and unilaterally in 12 patients. A chest tube was inserted in 13/21 patients (61.9%) with pneumothorax, 4/12 (33.3%) with pneumomediastinum without pneumothorax, and 5/14 (35.7%) with subcutaneous emphysema without pneumothorax. A chest tube was also inserted in 4/432 patients (0.9%) without barotrauma, mainly for pleural effusion. Renal replacement therapy was instituted in 15/49 patients (30.6%) with barotrauma and 86/432 patients (19.9%) without barotrauma (*p*=0.08).

### 3.4. Outcomes

The median duration of mechanical ventilation for all patients was 11 days (interquartile range: 5, 18 days); 16 days (interquartile range: 9, 30 days) for patients with barotrauma; and 10 days (interquartile range: 5, 18 days; *p* < 0.001) for those without barotrauma. Tracheostomy was also performed more frequently in patients with barotrauma (*p*=0.016). The lengths of stay in the ICU and hospital were significantly longer in patients with barotrauma compared to those without barotrauma (Table 3).

The overall mortality in the ICU was 30.8% and in the hospital, 40.3%. The ICU and hospital mortality rates of patients who developed barotrauma were significantly higher than those who did not (59.2% versus 27.5% for ICU mortality, *p* < 0.001, and 69.4% versus 37.0% for hospital mortality, *p* < 0.001) (Table 3).

On multivariable logistic regression analysis (Table 4), in which age, comorbid conditions such as obesity, hypertension, diabetes, asthma, chronic obstructive pulmonary disease, interstitial lung disease, heart failure, chronic kidney disease, stroke, cancer hyperlipidemia, arthritis, IMV, systemic steroids, and barotrauma were entered in the model as independent variables and age (OR: 1.050, 95% CI: 1.029–1.071), IMV (OR: 14.646, 95% CI: 8.238–26.037), barotrauma (OR: 2.784, 95% CI: 1.310–5.918), and asthma (OR: 0.468, 95% CI: 0.222–0.990) were significantly associated with hospital mortality.

The characteristics and management of patients with barotrauma who survived and did not survive are shown in Table 5. Nonsurvivors had a higher BMI and lymphocyte count, had less asthma as a comorbidity, and received less high-flow nasal oxygen and more IMV with a higher fraction of inspired oxygen on the first day of IMV.

## 4. Discussion

Our study found that pulmonary barotrauma occurred in 49 out of 481 patients (10.2%) with critical COVID-19 on a median of 4 days after ICU admission; pulmonary barotrauma was more common in patients who required artificial ventilation, especially IMV; most patients with pulmonary barotrauma received conservative treatment without chest tube insertion, especially in patients who did not have pneumothorax; pulmonary barotrauma was associated with a worse outcome and was an independent risk factor for hospital mortality.

In our study, 49 out of the 481 patients developed barotrauma in the form of pneumothorax, pneumomediastinum, and subcutaneous emphysema. This is slightly lower than the rate (15.6%) observed in a recent meta-analysis among critically ill patients with COVID-19 [5]. The prevalence of pulmonary barotrauma was the lowest in patients who did not require artificial ventilation (2.9%, 95% CI: 0.4–10.1%), higher in patients who were treated with NIV (without intubation) (3.0%, 95% CI: 0.8–7.6%), and highest in patients who required IMV (15.4%, 95% CI: 11.3–20.1%). This was observed in other studies [9]. COVID-19 itself may increase the risk of barotrauma. A systematic review of COVID-19 patients receiving mechanical ventilation (13 observational studies, 1814 patients) found that 14.7% had at least one barotrauma event compared with 6.3% of patients with non-COVID acute respiratory distress syndrome [7]. Data from randomized controlled trials on patients with acute respiratory distress syndrome (2468 patients) showed an incidence rate of barotrauma of 6–8% [13, 14].

The pathophysiology of barotrauma in COVID-19 is not very clear. A study investigated the radiographic patterns of barotrauma in patients with COVID-19 and observed that 41/43 patients (95%) demonstrated concurrent pneumomediastinum and subcutaneous emphysema or pneumomediastinum alone as the initial abnormal air collection [15]. The investigators concluded that this was consistent with pulmonary interstitial emphysema, where increased intrathoracic pressure causes overinflation of alveoli without adequate expansion of the associated vessel resulting in alveolar rupture and dissection of air into the broncho-vascular sheath and then dissection into the mediastinum, pleural space, subcutaneous tissues, and retroperitoneum [15, 16].

In the current study, we did not observe any significant association of comorbidities with pulmonary barotrauma. In contrast, hypertension and diabetes have been associated with barotrauma [17]. In our study, hemoglobin and lymphocyte count were slightly higher in barotrauma patients on univariate analysis. On multivariate analysis, the studied inflammatory markers were not associated with barotrauma. Other studies observed that certain but not all inflammatory markers were significantly elevated in patients with barotrauma [17, 18], with lymphocyte count being the only inflammatory marker associated with barotrauma on multivariate logistic regression [17]. We observed no association between NIV (without subsequent IMV) and the development of barotrauma. Similar findings were seen in another study [17]. IMV was significantly associated with pulmonary barotrauma in the multivariable regression analysis in our study. This was also observed in another study [18]. Surprisingly, Hamouri et al. noted that IMV was associated with less barotrauma [17]. We also observed no relationship between early ventilator settings, including PEEP, and barotrauma, likely because most of the study patients received lung protective strategies. While Protti et al. showed that a higher PEEP was a risk for barotrauma [19], a survey of 38 Italian hospitals found that pulmonary barotrauma that occurred with ventilatory settings that may be considered nonprotective was relatively uncommon [20]. For example, when the plateau airway pressure was >35 cm H_2_O, 2/113 (2%) patients had barotrauma, and when the tidal volume was >8 ml/kg of ideal body weight and the plateau airway pressure was >30 cm H_2_O, 12/134 (9%) patients had barotrauma [20]. Hence, SARS-CoV-2 itself may play an important role in the pathophysiology of barotrauma. It may be speculated that SARS-CoV-2 infection leads to frail alveoli by direct and/or indirect (inflammatory) alveolar injury, thus reducing epithelial-interstitial integrity [21, 22]. A rise in transpulmonary pressure, which can be precipitated by severe cough, respiratory distress, patient self-inflicted lung injury [23, 24], and/or positive pressure ventilation and would not affect normal alveoli, can be beyond the stress-strain threshold for the epithelial-interstitial integrity and lead to the rupture of the frail alveoli and thus interstitial emphysema [22]. Our finding of a slightly higher rate of barotrauma in patients who received IMV after failure of high-flow nasal oxygen and/or NIV (17.8% versus 9.6% for patients who only received IMV, *p* = 0.09) suggests that failure of noninvasive ventilatory support may increase the risk of barotrauma, possibly through patient self-inflicted lung injury, which may not have been fully mitigated by the noninvasive ventilatory support. Further studies are needed to understand the precise pathophysiology of barotrauma in COVD-19 and the role of the different inflammatory mediators.

We also found that patients with barotrauma had more respiratory bacterial cultures, likely because most received IMV, which would facilitate taking a deep tracheal aspirate for culture. Normal respiratory flora grew more often in patients with barotrauma. Whether bacterial coinfection or superinfection increases the risk of barotrauma in patients with COVID-19 is not clear and requires further analysis.

In the current study, chest tube insertion was performed in only 21/49 patients (42.9%) with barotrauma. This might be in part due to the occurrence of isolated pneumomediastinum or subcutaneous emphysema without pneumothorax. A study reported that 39/51 patients (76.5%) with pulmonary barotrauma were treated with a chest tube [17].

Barotrauma was associated with worse outcomes in our study, including a longer duration of IMV, a longer stay in the ICU, and higher mortality. These findings were also observed in other studies [18, 25]. Additionally, the rates of ICU and hospital mortality were significantly higher in patients with barotrauma compared with those without barotrauma, and the multivariable regression analysis showed that barotrauma was associated with an almost threefold increased risk of death (OR: 2.784, 95% CI: 1.310–5.918). Higher mortality with barotrauma was also observed in other studies [5, 18, 25]. Whether the increased mortality was solely due to barotrauma or that barotrauma indicated a more severe respiratory illness is not clear.

The results of this study should be interpreted based on its strengths and limitations. The strengths include the relatively large sample size and the inclusion of patients receiving different forms of oxygen therapy. The limitations include the retrospective design at a single center, which will limit its generalizability. The prevalence of barotrauma was below what was assumed for sample size calculation, which reduced the study's power to detect significant risk factors for barotrauma. Moreover, the presence of barotrauma was only assessed during the first 10 days of the ICU stay, and so more events may have occurred later; therefore, the prevalence may have been underestimated. However, we believe that most barotrauma occurs early during critical COVID-19. Other limitations include the unavailability of the levels of important inflammatory markers, such as interleukins, and the lack of assessment for acute respiratory distress syndrome and of periodic follow-ups. We should note that the association between barotrauma and IMV does not imply causality. It might be related to an unmeasured confounder and may be related to the severity of the respiratory disease leading to barotrauma, requirement of mechanical ventilation, and increased mortality at the same time.

## 5. Conclusions

Barotrauma was common in patients with critical COVID-19, especially in those receiving IMV. Barotrauma was associated with worse outcomes, including mortality. Whether barotrauma itself worsens outcomes or whether its occurrence is a marker of more severe illness, it remains unclear. Caution during intubation, by avoiding aggressive Ambu bagging, and the implementation of lung protective strategies during NIV and IMV are warranted when caring for patients with COVID-19 and severe hypoxemic respiratory failure.

## Figures and Tables

**Table 1:** Baseline characteristics of patients with critical COVID-19.

Variable	All patients *N* = 481		Barotrauma *N* = 49	No barotrauma *N* = 432	*P* value
Age (years), mean ± SD	61.3 ± 14.8		63.5 ± 13.7	61.0 ± 14.9	0.26
Sex, *N* (%)					
Males	346 (71.9)		37 (75.5)	309 (71.5)	0.557
Females	135 (28.1)		12 (24.5)	123 (28.5)	
Body mass index (kg/m^2^), median (*Q*1, *Q*3)	30.1 (25.8, 34.6)		30.9 (26.6, 35.8)	30.1 (25.7, 34.4)	0.489
Smoking, *N* (%)	6 (1.2)		0 (0.0)	6 (1.4)	0.566
Comorbidities					
Obesity, *N* (%)		90 (18.7)	6 (12.2)	84 (19.4)	0.221
Hypertension, *N* (%)		324 (67.4)	35 (71.4)	289 (66.9)	0.522
Diabetes, *N* (%)		398 (82.7)	45 (91.8)	353 (81.7)	0.076
Chronic kidney disease, *N* (%)		66 (13.7)	5 (10.2)	61 (14.1)	0.450
Hyperlipidaemia, *N* (%)		164 (34.1)	13 (26.5)	151 (35.0)	0.238
Chronic obstructive pulmonary disease, *N* (%)		8 (1.7)	1 (2.0)	7 (1.6)	0.579
Asthma, *N* (%)		54 (11.2)	8 (16.3)	46 (10.6)	0.233
Previous pneumothorax, *N* (%)		0 (0.0)	0 (0.0)	0 (0.0)	—
Interstitial lung disease, *N* (%)		4 (0.8)	1 (2.0)	3 (0.7)	0.350
Stroke, *N* (%)		28 (5.8)	2 (4.1)	26 (6.0)	0.757
Heart failure, *N* (%)		48 (10.0)	4 (8.2)	44 (10.2)	0.805
Non-alcoholic fatty liver disease, *N* (%)		6 (1.2)	0 (0.0)	6 (1.4)	1.000
Arthritis, *N* (%)		48 (10.0)	5 (10.2)	43 (10.0)	1.000
Cancer, *N* (%)		18 (3.7)	0 (0.0)	18 (4.2)	0.239
Depression, *N* (%)		25 (5.2)	1 (2.0)	24 (5.6)	0.497
Laboratory findings					
White blood cell count (×10^9^/L) mean ± SD		10.5 ± 5.3	11.0 ± 4.8	10.5 ± 5.3	0.548
Neutrophil count (×10^9^/L) mean ± SD		7.9 ± 3.7	8.6 ± 3.3	7.8 ± 3.7	0.273
Lymphocyte count (×10^9^/L), median (*Q*1, *Q*3)		0.9 (0.7, 1.2)	0.8 (0.6, 1.0)	0.7 (0.9, 1.2)	0.067
Hemoglobin (g/L) mean ± SD		126.8 ± 21.2	134.9 ± 27.9	125.8 ± 20.1	0.032
Platelets (×10^9^/L) mean ± SD		269.5 ± 110.6	252.9 ± 108.1	271.4 ± 110.9	0.267
Creatinine (*μ*mol/L), median (*Q*1, *Q*3)		86.0 (70.0, 132.8)	89.0 (68.0, 112.5)	86.0 (70.0, 135.5)	0.762
Lactate (mmol/L) median (*Q*1, *Q*3)		1.8 (1.3, 2.6)	1.9 (1.6, 2.4)	1.7 (1.3, 2.6)	0.152
Fibrinogen (g/L) mean ± SD		5.59 ± 1.85	5.42 ± 1.98	5.62 ± 1.84	0.622
D-dimer (*μ*g/mL) median (*Q*1, *Q*3)		1.18 (0.72, 3.26)	1.90 (0.88, 4.51)	1.15 (0.71, 2.97)	0.101
Ferritin (*μ*g/L) median (*Q*1, *Q*3)		770.6 (389.2, 2001.9)	710.1 (384.3, 2121.2)	773.4 (390.5, 1996.8)	0.918
CXR findings on ICU admission, *N* (%)					
Unilateral lung infiltrates		33 (6.9)	3 (6.1)	30 (6.9)	1.0
Bilateral lung infiltrates		448 (93.1)	46 (93.9)	402 (93.1)	
Management of COVID-19					
Systemic corticosteroids, *N* (%)		388 (80.7)	43 (87.8)	345 (79.9)	0.185
IJ central line, *N* (%)		219 (45.5)	37 (75.5)	182 (42.1)	<0.0001
Subclavian central line, *N* (%)		10 (2.1)	0 (0.0)	10 (2.3)	0.609
No artificial (non-invasive or invasive) ventilation		69 (14.3)	2 (4.1)	67 (15.5)	0.031
High flow nasal oxygen, *N* (%)		294 (61.1)	29 (59.2)	265 (61.3)	0.769
Non-invasive ventilation, *N* (%)		302 (62.8)	34 (69.4)	268 (62.0)	0.313
No intubation		132 (27.4)	4 (8.2)	128 (29.6)	0.001
Intubation/invasive ventilation, *N* (%)		280 (58.2)	43 (87.8)	237 (54.9)	0.0001
Ventilator settings (day 1)					
Lowest FiO_2_, mean ± SD		41.9 ± 11.9	44.7 ± 14.7	41.5 ± 11.5	0.088
Maximum PEEP (cm H_2_O), mean ± SD		12.0 ± 2.9	11.6 ± 2.2	12.1 ± 3.0	0.264
Maximum tidal volume (ml), mean ± SD		398.9 ± 53.7	407.0 ± 49.0	397.3 ± 54.4	0.281
Tidal volume (ml) per kg ideal body weight, median (*Q*1, *Q*3)		6.7 (6.0, 7.4)	6.8 (6.0, 7.5)	6.6 (6.1, 7.3)	0.857
Maximum peak airway pressure (cm H_2_O) mean ± SD		32.7 ± 6.1	31.5 ± 4.9	32.9 ± 6.3	0.111
Maximum plateau pressure (cm H_2_O), mean ± SD		29.4 ± 5.1	27.3 ± 5.1	29.8 ± 5.1	0.058

PEEP: positive end-expiratory pressure, SD: standard deviation, IJ: internal jugular, FiO_2_: fraction of inspired oxygen, *Q*1: first quartile, and *Q*3: third quartile.

**Table 2:** Multivariable logistic regression analysis for the risk factors of pulmonary barotrauma. Variables entered in the model are obesity, diabetes, asthma, cancer, lymphocyte count, lactate, d-dimer, corticosteroid treatment, hyperlipidemia, and invasive mechanical ventilation.

Variable	Odds ratio	95% confidence interval		*P* value
Invasive mechanical ventilation	14.558	1.833	115.601	0.011
Steroid treatment	1.551	0.305	7.887	0.597
Diabetes	1.191	0.212	6.683	0.842
Asthma	1.132	0.200	6.404	0.888
D-dimer level per unit increment	0.967	0.875	1.069	0.510
Lactate level per unit increment	0.895	0.655	1.223	0.487
Hyperlipidemia	0.813	0.259	2.553	0.723
Obesity	0.673	0.193	2.345	0.534
Lymphocyte count per unit increment	0.465	0.130	1.663	0.239
Cancer	0.000	0.000	—	0.999

**Table 3:** The outcomes of patients with critical COVID-19 categorized by the occurrence of pulmonary barotrauma.

Variable	All patients *N* = 481	Barotrauma *N* = 49	No barotrauma *N* = 432	*P* value
Tracheostomy, *N* (%)	45 (9.4)	10 (20.4)	35 (8.1)	0.016
ICU mortality, *N* (%)	148 (30.8)	29 (59.2)	119 (27.5)	<0.0001
Hospital mortality, *N* (%)	194 (40.3)	34 (69.4)	160 (37.0)	<0.0001
Length of stay in ICU, median (*Q*1, *Q*3)	10.0 (5.0, 19.0)	17.0 (11.0, 29.0)	9.0 (5.0, 18.0)	<0.0001
Length of stay in hospital, median (*Q*1, *Q*3)	17.0 (12.0, 28.0)	23.0 (16.5, 38.0)	17.0 (11.0, 27.0)	<0.0001
Duration of IMV⁣^*∗*^, median (*Q*1, *Q*3)	11.0 (5.0, 18.0)	16.0 (9.0, 30.0)	10.0 (5.0, 18.0)	<0.0001

⁣^*∗*^Calculated for patients who received invasive mechanical ventilation. IMV: invasive mechanical ventilation, ICU: intensive care unit, *Q*1: first quartile, and *Q*3: third quartile.

**Table 4:** Multivariable logistic regression analysis for the risk factors of hospital mortality. Variables entered in the model include obesity, corticosteroids, invasive mechanical ventilation, age, hypertension, diabetes, chronic kidney disease, interstitial lung disease, stroke, heart failure, barotrauma, chronic obstructive pulmonary disease, asthma, nonalcoholic fatty liver disease, arthritis, and cancer.

Variable	Odds ratio	95% confidence interval		*P* value
Invasive mechanical ventilation	14.646	8.238	26.037	0.0001
Chronic obstructive pulmonary disease	6.606	0.977	44.678	0.053
Non-alcoholic fatty liver disease	3.174	0.389	25.868	0.281
Interstitial lung disease	2.802	0.168	46.713	0.473
Barotrauma	2.784	1.310	5.918	0.008
Stroke	1.774	0.655	4.806	0.260
Arthritis	1.744	0.778	3.908	0.177
Cancer	1.581	0.489	5.108	0.444
Heart failure	1.568	0.710	3.459	0.266
Obesity	1.097	0.604	1.992	0.761
Steroid treatment	0.593	0.326	1.079	0.087
Chronic kidney disease	1.239	0.626	2.449	0.538
Age per one year increment	1.050	1.029	1.071	0.000
Asthma	0.468	0.222	0.990	0.047
Diabetes	0.748	0.346	1.615	0.459
Hypertension	0.558	0.312	1.000	0.050

**Table 5:** Characteristics of the 49 patients with pulmonary barotrauma who survived to hospital discharge and died in the hospital.

Variable	Survived *N* = 15	Died *N* = 34	*P* value
Age (years), mean ± SD	60.7 ± 16.2	64.8 ± 12.6	0.337
Sex, *N* (%)			
Males	13 (86.7)	24 (70.6)	0.298
Females	2 (13.3)	10 (29.4)	
Body mass index (kg/m^2^), median (*Q*1, *Q*3)	25.9 (24.5, 30.9)	32.7 (28.0, 37.5)	0.005
Comorbidities			
Obesity, *N* (%)	2 (13.3)	4 (11.8)	1.0
Hypertension, *N* (%)	12 (80.0)	23 (67.6)	0.502
Diabetes, *N* (%)	14 (93.3)	31 (91.2)	1.0
Chronic kidney disease, *N* (%)	1 (6.7)	4 (11.8)	1.0
Hyperlipidemia, *N* (%)	5 (33.3)	8 (23.5)	0.500
Chronic obstructive pulmonary disease, *N* (%)	0 (0)	1 (2.9)	1.0
Asthma, *N* (%)	5 (33.3)	3 (8.8)	0.047
Previous pneumothorax, *N* (%)	0 (0.0)	0 (0.0)	—
Interstitial lung disease, *N* (%)	1 (6.7)	0 (0)	0.306
Stroke, *N* (%)	1 (6.7)	1 (2.9)	0.523
Heart failure, *N* (%)	3 (20.0)	1 (2.9)	0.079
Non-alcoholic fatty liver disease, *N* (%)	0 (0.0)	0 (0.0)	—
Arthritis, *N* (%)	0 (0.0)	5 (14.7	0.306
Cancer, *N* (%)	0 (0.0)	0 (0.0)	—
Depression, *N* (%)	1 (2.0)	1 (2.9)	1.0
Laboratory findings			
White blood cell count (×10^9^/L) mean ± SD	11.6 ± 5.0	10.7 ± 4.8	0.520
Neutrophil count (×10^9^/L) mean ± SD	9.4 ± 3.3	8.2 ± 3.3	0.341
Lymphocyte count (×10^9^/L), median (*Q*1, *Q*3)	0.6 (0.6, 0.7)	0.9 (0.7, 1.1)	0.025
Hemoglobin (g/L) mean ± SD	134.5 ± 21.4	135.0 ± 30.6	0.949
Platelets (×10^9^/L) mean ± SD	246.1 ± 97.4	255.8 ± 113.8	0.776
Creatinine (*μ*mol/L), median (*Q*1, *Q*3)	81.0 (67.0, 111.0)	91.0 (68.0, 123.0)	0.632
Lactate (mmol/L) median (*Q*1, *Q*3)	1.9 (1.6, 2.6)	1.9 (1.3, 2.6)	0.921
Fibrinogen (g/L) mean ± SD	4.35 ± 1.76	5.63 ± 2.00	0.247
D-dimer (*μ*g/mL) median (*Q*1, *Q*3)	3.63 (1.14, 4.64)	1.90 (0.76, 6.35)	0.556
Ferritin (*μ*g/L) median (*Q*1, *Q*3)	894.0 (497.7, 3792.61)	673.0 (370.4, 1362.4)	0.296
Management of COVID-19			
Systemic corticosteroids, *N* (%)	12 (80.0)	31 (91.2)	0.353
High flow nasal oxygen, *N* (%)	12 (80.0)	17 (50.0)	0.049
Non-invasive ventilation, *N* (%)	9 (60.0)	25 (73.5)	0.502
Intubation/invasive ventilation, *N* (%)	10 (66.7)	33 (97.1)	0.008
Ventilator settings (day 1)			
Lowest FiO_2_, mean ± SD	35.8 ± 10.1	47.5 ± 15.4	0.031
Maximum PEEP (cmH_2_O), mean ± SD	10.8 ± 2.3	11.8 ± 2.2	0.214
Maximum tidal volume (ml), mean ± SD	405.0 ± 44.8	407.6 ± 50.8	0.886
Tidal volume (ml) per kg ideal body weight, median (*Q*1, *Q*3)	6.5 (6.0, 6.9)	7.0 (6.0, 7.6)	0.358
Maximum peak airway pressure (cm H_2_O) mean ± SD	30.8 ± 4.0	31.8 ± 5.2	0.612
Maximum plateau pressure (cm H_2_O), mean ± SD	24.7 ± 5.9	27.8 ± 5.0	0.341
Type of barotrauma, *N* (%)			
Pneumothorax	10 (66.7)	11 (32.4)	0.025
Pneumomediastinum	9 (60.0)	16 (47.1)	0.404
Subcutaneous emphysema	10 (66.7)	15 (44.1)	0.146

PEEP: positive end-expiratory pressure, SD: standard deviation, IJ: internal jugular, FiO_2_: fraction of inspired oxygen, *Q*1: first quartile, and *Q*3: third quartile.

## Data Availability

The data used to support the findings in this study are available from the corresponding author on request. The data are not publicly available due to institutional policies of maintaining the confidentiality of patient data.

## Abbreviations

BMI: Body mass index
CI: Confidence interval
COVID-19: Coronavirus disease 2019
FiO_2_: Fraction of inspired oxygen
ICU: Intensive care unit
IJ: Internal jugular
IMV: Invasive mechanical ventilation
NIV: Non-invasive ventilation
OR: Odds ratio
PEEP: Positive end-expiratory pressure
Q1: First quartile
Q3: Third quartile
SARS-CoV-2: Severe acute respiratory syndrome coronavirus 2
SD: Standard deviation.

## References

[1] Wang D., Hu B., Hu C. (2020). Clinical characteristics of 138 hospitalized patients with 2019 novel coronavirus–infected pneumonia in Wuhan, China. *JAMA*.

[2] Yang X., Yu Y., Xu J. (2020). Clinical course and outcomes of critically ill patients with SARS-CoV-2 pneumonia in Wuhan, China: a single-centered, retrospective, observational study. *The Lancet Respiratory Medicine*.

[3] Huang C., Wang Y., Li X. (2020). Clinical features of patients infected with 2019 novel coronavirus in Wuhan, China. *Lancet*.

[4] Chen N., Zhou M., Dong X. (2020). Epidemiological and clinical characteristics of 99 cases of 2019 novel coronavirus pneumonia in Wuhan, China: a descriptive study. *Lancet*.

[5] Shrestha D. B., Sedhai Y. R., Budhathoki P. (2022). Pulmonary barotrauma in COVID-19: a systematic review and meta-analysis. *Annals of Medicine and Surgery*.

[6] McGuinness G., Zhan C., Rosenberg N. (2020). Increased incidence of barotrauma in patients with COVID-19 on invasive mechanical ventilation. *Radiology*.

[7] Belletti A., Todaro G., Valsecchi G. (2022). Barotrauma in coronavirus disease 2019 patients undergoing invasive mechanical ventilation: a systematic literature review. *Critical Care Medicine*.

[8] Knox D. B., Brunhoeber A., Peltan I. D., Brown S. M., Lanspa M. J. (2022). Comparison of radiographic pneumothorax and pneumomediastinum in COVID-19 vs. non-COVID-19 acute respiratory distress syndrome. *Intensive Care Medicine*.

[9] Rajdev K., Spanel A. J., McMillan S. (2021). Pulmonary barotrauma in COVID-19 patients with ARDS on invasive and non-invasive positive pressure ventilation. *Journal of Intensive Care Medicine*.

[10] Al-Dorzi H. M., Aldawood A. S., Almatrood A. (2021). Managing critical care during COVID-19 pandemic: the experience of an ICU of a tertiary care hospital. *Journal of Infection and Public Health*.

[11] National Institutes of Health (2020). Clinical spectrum of SARS-CoV-2 infection. https://www.covid19treatmentguidelines.nih.gov/overview/clinical-spectrum/.

[12] Bursac Z., Gauss C. H., Williams D. K., Hosmer D. W. (2008). Purposeful selection of variables in logistic regression. *Source Code for Biology and Medicine*.

[13] Kang H., Yang H., Tong Z. (2019). Recruitment manoeuvres for adults with acute respiratory distress syndrome receiving mechanical ventilation: a systematic review and meta-analysis. *Journal of Critical Care*.

[14] Shao S., Kang H., Tong Z. (2020). Early neuromuscular blocking agents for adults with acute respiratory distress syndrome: a systematic review, meta-analysis and meta-regression. *BMJ Open*.

[15] Steinberger S., Finkelstein M., Pagano A. (2022). Barotrauma in COVID 19: incidence, pathophysiology, and effect on prognosis. *Clinical Imaging*.

[16] Kouritas V. K., Papagiannopoulos K., Lazaridis G. (2015). *Journal of Thoracic Disease*.

[17] Hamouri S., Samrah S. M., Albawaih O. (2021). Pulmonary barotrauma in COVID-19 patients: invasive versus noninvasive positive pressure ventilation. *International Journal of General Medicine*.

[18] Elsaaran H., AlQinai S., AlTarrah D. (2021). Prevalence and risk factors of barotrauma in Covid-19 patients admitted to an intensive care unit in Kuwait; a retrospective cohort study. *Annals of Medicine and Surgery*.

[19] Protti A., Greco M., Filippini M., Vilardo A. M., Langer T., Tavola M. (2020). Barotrauma in mechanically-ventilated patients with coronavirus disease 2019: a survey of 38 hospitals in lombardy italy. *Minerva anestesiologica*.

[20] Protti A., Greco M., Filippini M. (2021). Barotrauma in mechanically ventilated patients with Coronavirus disease 2019: a survey of 38 hospitals in Lombardy, Italy. *Minerva Anestesiologica*.

[21] Borczuk A. C., Salvatore S. P., Seshan S. V. (2020). COVID-19 pulmonary pathology: a multi-institutional autopsy cohort from Italy and New York City. *Modern Pathology*.

[22] Somasundram K., Agbontaen K., Singh S. (2021). Pneumomediastinum in COVID-19: merely a matter of lung frailty?. *Respiration*.

[23] Marini J. J., Gattinoni L. (2020). Management of COVID-19 respiratory distress. *JAMA*.

[24] Grieco D. L., Maggiore S. M., Roca O. (2021). Non-invasive ventilatory support and high-flow nasal oxygen as first-line treatment of acute hypoxemic respiratory failure and ARDS. *Intensive Care Medicine*.

[25] Venkateswaran V., Soni K., Chaturvedi Chaturvedi A. (2022). Barotrauma in critically ill COVID-19 patients: a retrospective case-control study. *Anaesthesiology Intensive Therapy*.

